# Executive, behavioural and emotional functioning in Spanish children with dyslexia

**DOI:** 10.1590/2317-1782/e20240352en

**Published:** 2025-12-12

**Authors:** Miguel López-Zamora, Nadia Porcar-Gozalbo, Alejandro Cano-Villagrasa, Isabel López-Chicheri

**Affiliations:** 1 Departamento de Psicología Evolutiva y de la Educación, Facultad de Psicología y Logopedia, Universidad de Málaga – UMA - Málaga (Andalucia), España.; 2 Facultad de Ciencias de la Salud, Universidad Internacional de Valencia – VIU - Valencia (Comunidad Valenciana), España.; 3 Universidad Católica de Murcia – UCAM - Murcia (Región de Murcia), España.

**Keywords:** Dyslexia, Executive Functions, Anxiety, Depression, Behavioral Disorder

## Abstract

**Purpose:**

Dyslexia is a specific learning disorder that affects reading and is associated with emotional and behavioral problems. Previous research indicates that children with dyslexia are at greater risk of developing anxiety, depression, and difficulties in executive functions, which affect their academic performance and well being.

**Methods:**

This study explored and compared behavioral, emotional, and executive functioning profiles in children with dyslexia and in neurotypical children in a sample of 120 children aged 8 to 10, divided into a dyslexia group and a control group. The BASC-3, SENA, and ENFEN were used for assessments, and data were analyzed using t-tests, Levene’s test, and mediation analyses.

**Results:**

Children with dyslexia showed significantly higher levels of aggressiveness, anxiety, depression, and attention problems. In addition, they exhibited difficulties in executive functions such as resistance to interference and verbal fluency, highlighting the impact of dyslexia in these areas.

**Conclusion:**

Mediation analyses suggest that dyslexia is a potential indicator of difficulties in executive functioning and behavior, as well as influencing internalized and externalized emotional problems. These findings underscore the need to implement comprehensive educational and therapeutic strategies to address the needs of this vulnerable population.

## Introduction

The development of specific learning processes for reading represents a significant challenge for children's maturational and academic trajectory, constituting a determining factor in educational success during the early school years^(1,2)^. Unlike language, reading is a relatively recent neurocognitive process that does not have a predetermined anatomical basis in the brain, which explains the need for explicit instruction for its acquisition^(3-5)^. This learning requires the precise organization of the cortical system to effectively integrate the various reading processes. However, several factors can lead to ineffective cortical connections, resulting in difficulties in the acquisition and development of reading skills^(6-8)^.

Dyslexia is one of the most prevalent disorders affecting reading acquisition. It is a specific learning disorder that affects between 4.66% and 9.22% of school-age children in Europe^(9,10)^. Globally, a systematic review conducted by Yang et al.^(11)^, which included 58 studies published from the 1950s to 2021, revealed a combined prevalence of 7.1%. This review, covering schoolchildren aged 6-13 years in 16 countries, including six low-income countries, showed similar prevalence estimates in high-income countries, ranging from 6.8% to 8.3%. These data highlight the importance of studying and understanding the neurocognitive bases of reading acquisition, as well as the factors influencing its development to adequately address the effects of dyslexia.

However, despite progress in characterizing the neurocognitive profiles associated with dyslexia, a significant gap in the literature regarding an integrated understanding of the emotional, behavioral, and executive factors involved remains^(7,8)^. Most studies address these dimensions in isolation without sufficiently exploring the possible mediating relationships between them^(10,11)^. In particular, the role of executive functions as potential explanatory mechanisms linking reading difficulties with emotional and behavioral symptoms remains underexplored, limiting both the interpretation of findings and the development of more specific and effective interventions^(11)^.

Despite having normative intelligence and no neurological or sensory deficits hindering this skill, children with dyslexia present difficulties in word recognition, spelling, and decoding^(12,13)^. Therefore, learning to read represents a significant, constant, and frustrating limitation for children with dyslexia, negatively impacting their lives^(14)^.

Recent research has reported that nearly 60% of children with a primary diagnosis of dyslexia meet the criteria for at least one mood, anxiety, or depressive disorder^(15-20)^. Moreover, a high proportion of anxiety and depressive disorders among children with dyslexia. Burke et al.^(21)^ showed that individuals with reading process difficulties exhibit significantly higher rates of internalizing and externalizing disorders than individuals without reading impairments. Similarly, Wang^(22)^ showed that children with dyslexia who have higher levels of anxiety and depression perform worse in academic learning skill.

A lower academic performance predicts an increase in anxiety and depressive symptoms in children with dyslexia^(23)^. Studies have identified that school-related stress and anxiety are more prevalent in primary school students with dyslexia^(24)^ compared to students at higher academic levels, such as university students^(25)^. Because children with dyslexia perceive themselves as worse at academic tasks, they often develop disruptive behaviors and social isolation in classrooms^(26)^, which reduces their self-esteem and distorts their self-concept^(27)^. According to authors such as Alexander-Passe^(28)^, these disruptive behaviors are triggered by anxiety, which in turn is produced by anxious feelings, leading children with dyslexia to frequently interrupt class, ignore the teacherâ€™s explanations, or get into fights with classmates.

Similarly, low self-esteem may cause depression in children with dyslexia^(20,29)^. However, the literature results are inconsistent. Internalizing symptoms refer to emotional difficulties that manifest internally, such as anxiety, depression, social isolation, or low self-esteem. These problems are usually less visible to others but deeply affect a personâ€™s emotional well being. On the other hand, externalizing symptoms involve behaviors that manifest outwardly, such as aggression, impulsivity, or disruptive conduct, and are more noticeable due to their impact on the environment. In this context, studies by Katsantonis et al.^(30)^ and Miller et al.^(31)^ have not found a significant relationship between dyslexia and elevated levels of internalizing symptoms, such as anxiety or depression, suggesting that the emotional difficulties experienced by these children may not be as evident in terms of internalized symptoms. Therefore, no global consensus has clearly described the relationship between bullying, victimization, and the onset of anxiety and mood disorders.

Apart from evidence on the emotional alterations of individuals with specific reading learning difficulties, the specific executive functioning profile in this population may be closely related to anxiety, depression, and behavioral disorders^(32)^. Children diagnosed with dyslexia present deficits in executive functioning^(33-35)^, regardless of additional difficulties that may arise or develop as comorbid conditions^(36,37)^. Consistent empirical evidence indicates that children with dyslexia exhibit poor performance in executive function tasks involving motivational and emotional processes^(38)^.

There is also deterioration in functions such as behavioral inhibition, which is associated with aggressive behavior and conduct disorder in primary school children (6-12 years) and preschoolers (4 years)^(39)^, compared to other functions such as planning and working memory. However, the scientific literature does not reveal a common pattern in executive function impairments among children with dyslexia. This is largely due to participant heterogeneity and individual differences, creating a complex situation for comparing results across published studies.

In this regard, a better understanding of executive functioning and its role in dyslexia has not only theoretical implications but also high applied value. Identifying specific executive impairment profiles could enable the design of more personalized intervention strategies that address not only reading difficulties but also the associated emotional and behavioral symptoms. This is especially relevant for health and education professionals as it could facilitate the implementation of preventive and therapeutic programs in school contexts, promoting an interdisciplinary and needs-centered approach for students with dyslexia.

Therefore, the main objective of this study was to explore differences in behavioral processes, the pattern of internalizing and externalizing emotional behaviors, as well as executive functioning in a cohort of children with dyslexia, comparing the measurement results in each of the areas with another group of children with typical development. Likewise, the relationship between all these variables and their mediating effects was explored.

## Method

### Participants

A total of 120 participants (58 girls and 62 boys) aged between 8 and 10 years (M = 9.2) were selected. Participants diagnosed with dyslexia had been evaluated by their reference hospital and educational center, following the diagnostic criteria established in the DSM-5 TR^(9)^. The assessment tool used by healthcare and educational professionals (psychologists and speech-language therapists) for diagnosing dyslexia was the PROLEC-R test^(40)^. The sample was divided into two groups: an experimental group composed of 60 individuals diagnosed with dyslexia (G-DYSLEXIA) and a comparison group consisting of 60 individuals with typical reading development (G-CONTROL). All study participants were monolingual Spanish speakers born in Spain.

A series of inclusion and exclusion criteria were applied to form the sample groups. The inclusion criteria for the G-DYSLEXIA group were as follows: diagnosis of dyslexia, age between 8 and 10 years, and possessing expressive language. The exclusion criteria for participants in both groups (G-DYSLEXIA and G-CONTROL) included having a severe sensory pathology, a diagnosis of intellectual disability, suspected severe psychiatric disorder, or other pre-existing conditions that could hinder the assessment.

### Instruments and materials

#### BASC-3, Behavior Assessment System for Children Third Edition

The BASC-3, Behavior Assessment System for Children – Third Edition (BASC-3)^(41)^, is a clinical tool used to assess the emotions and behaviors of children and adolescents, detecting maladaptive disorders in contexts such as family and school. The BASC-3 includes a self-report for the child and two questionnaires aimed at parents (P) and teachers or tutors (T). For the purposes of this research, only the questionnaires for parents and teachers were considered. The Cronbach’s α for this test is 0.90.

#### SENA, System for the Evaluation of Children and Adolescents

The SENA, System for the Evaluation of Children and Adolescents (SENA)^(42)^, consists of a set of nine questionnaires designed for three specific age groups: Preschool (3–6 years), Primary (6–12 years), and Secondary (12–18 years). This system includes questionnaires for gathering information from different informants in the main contexts in which the child interacts, such as family and school. It also incorporates three self-report models adapted to the child’s age, applicable from age 6 onwards. In each questionnaire, informants rate the frequency with which the described behavior occurs using a five-point scale (from Never or Almost Never to Always or Almost Always), except for the self-report for children aged 6–8 years, which uses a three-option scale: Yes, No, and Sometimes. The Cronbach’s α for this test is 0.81.

#### ENFEN, Neuropsychological Evaluation of Executive Functions in Children

The ENFEN, Neuropsychological Evaluation of Executive Functions in Children (ENFEN)^(43)^ is a tool designed to individually assess the level of maturity and cognitive performance in tasks related to executive functions in children aged −12 years. The battery consists of four tests (Verbal Fluency, Trail Construction, Ring Construction, and Resistance to Interference) that measure different aspects of executive functions. The results obtained allow for a deeper diagnosis and guide neuropsychological intervention, both in typically developing children and in those with developmental delays or cognitive or emotional alterations resulting from dysfunctions or brain damage. The Cronbach’s α for this test is 0.84.

### Procedure

The study was approved by the ethics committee of Universidad de Málaga (UMA). All families of the participants agreed to participate in the study by signing an informed consent form, ensuring anonymity and data protection. Data collection was conducted in two stages. An initial interview was conducted in the first stage, followed by the administration of behavioral, emotional, and executive functioning assessment tests. Two individual sessions were conducted, each lasting approximately 45 min, spaced 3 to 5 business days apart, depending on the availability of the school and of the child's needs. This interval helped prevent fatigue and ensured a more accurate and comfortable assessment for the participants.

The assessments were administered by a team of trained professionals consisting of educational psychologists and neuropsychologists with experience in child assessment. All evaluators received specific training for the standardized administration of each instrument to ensure the data reliability. In the second stage, an analysis of the data collected from the study participants were analyzed using the selected statistical methods and a database was generated with the results of the evaluated individuals.

### Design

This is a descriptive, cross-sectional study with an experimental group (G-DYSLEXIA) and a control group (G-CONTROL). The choice of a cross-sectional design, rather than a longitudinal one, responds to the need to evaluate significant differences at a specific point in early school development, a stage in which reading difficulties and their possible emotional and behavioral repercussions are most evident. This design allows for an efficient comparison of two well defined groups within a homogeneous age range, facilitating a more direct interpretation of results regarding the diagnosis of dyslexia.

The dependent variables were the functions and skills assessed using the aforementioned standardized instruments. The independent variable of the study was dyslexia diagnosis in this population.

To analyze the study variables, a normality test was first performed using the Chi-square test for the sociodemographic variables of the participants, such as Sex, Age, Diagnosis, Years of Treatment, Comorbidity, School Support, Gestational Weeks, and Apgar. To examine the relationships and trends among the variables related to behavioral, emotional, and executive functioning components, a descriptive statistical analysis was conducted along with a paired-samples t-test to observe differences between the two groups. Likewise, to observe the relationships and influence among the variables in the present study, a mediation analysis was performed between the variables related to emotional problems and dyslexia. All analyses were performed using the SPSS software, version 29.

## Results

Descriptive analysis was conducted on the sociodemographic variables of sex, age, dyslexia diagnosis, years of treatment, comorbidity, school support, gestational weeks, and Apgar score, along with the Chi-square test results for each variable. No significant differences were found in any of the variables (*P* > 0.05).

To address the objectives of this study, the results from the independent samples t-test analyses comparing the scores of the two groups on measures related to behavior, internalizing and externalizing emotional behavior, as well as executive functioning are presented below. Differences in scores were calculated using independent samples t-tests for the cognitive competence variables. The results revealed significant differences in all variables related to executive functioning. Levene’s test for equality of variances was then conducted for each variable, confirming the assumption of homogeneity of variances, as the significance value exceeded 0.05 (*P* > 0.05), indicating that the variances of the groups on the assessed variables were homogeneous (Tables 1, 2, and 3).

**Table 1:** Results of differences in measures related to the behavioral profile between the G-DYSLEXIA and G-CONTROL groups, assessed using the BASC-3

Behavioral and Emotional Problems	Groups		Levene's test for equality of variances			T test for equality of means				
	*G-DYSLEXIA*	*G-CONTROL*	*F*	*Sig.*	*η2*	*t*	*df*	*Sig. (bilateral)*	*Mean difference*	ð
**Composite, Clinical, and Adaptive Scales**										
*Aggressiveness*	M = 53.38 SD = 8.36	M = 17.63 SD = 5.91	14.878	<.001	0.586	27.022	118	<.001	35.750	7.246
*Anxiety*	M = 55.63 SD = 7.44	M = 19.37 SD = 6.49	1.254	0.265	0.689	28.417	118	<.001	36.267	6.990
*Depression*	M = 54.13 SD = 7.34	M = 19.63 SD = 6.56	0.698	0.405	0.521	27.134	118	<.001	34.500	6.964
*Somatization*	M = 52.28 SD = 7.70	M = 18.77 SD = 6.22	2.227	0.138	0.855	26.210	118	<.001	33.517	7.049
*Atypicality*	M = 54.95 SD = 8.10	M = 18.85 SD = 6.05	11.679	<.001	0.971	27.631	118	<.001	36.100	7.156
*Withdrawal*	M = 53.48 SD = 8.06	M = 19.93 SD = 5.85	10.826	0.001	0.685	26.066	118	<.001	33.550	7.050
*Attention Problems*	M = 53.28 SD = 7.61	M = 20.13 SD = 5.40	10.830	0.001	0.753	27.497	118	<.001	33.150	6.603
*Adaptability*	M = 55.08 SD = 8.12	M = 19.38 SD = 6.24	5.360	0.022	0.698	26.978	118	<.001	35.700	7.248
*Social Skills*	M = 55.33 SD = 8.54	M = 19.83 SD = 5.43	20.250	<.001	0.611	27.160	118	<.001	35.500	7.159
*Daily Activities*	M = 53.53 SD = 7.39	M = 19.57 SD = 5.59	3.038	0.084	0.684	28.361	118	<.001	33.967	6.560
*Functional Communication*	M = 54.45 SD = 8.23	M = 18.20 SD = 5.99	5.155	0.025	0.752	26.227	118	<.001	35.500	7.414
**Content Scales**										
*Anger Control*	M = 54.48 SD = 8.49	M = 18.20 SD = 5.99	8.091	0.005	0.814	27.039	118	<.001	36.283	7.350
*Bullying*	M = 53.55 SD = 7.05	M = 19.30 SD = 6.20	0.472	0.493	0.862	28.239	118	<.001	34.250	6.643
*Social Developmental Disorders*	M = 56.15 SD = 7.66	M = 19.42 SD = 5.92	5.693	0.019	0.834	29.368	118	<.001	36.733	6.851
*Emotional Self-Control*	M = 55.00 SD = 7.72	M = 19.32 SD = 5.98	7.390	0.008	0.812	28.293	118	<.001	35.683	6.908
*Executive Functioning*	M = 53.57 SD = 7.72	M = 17.73 SD = 6.46	3.045	0.084	0.877	27.560	118	<.001	35.833	7.122
*Negative Emotionality*	M = 55.88 SD = 7.45	M = 18.43 SD = 5.51	6.806	0.010	0.683	27.560	118	<.001	37.450	6.554
*Resilience*	M = 52.18 SD = 8.44	M = 19.23 SD = 5.89	6.490	0.012	0.509	31.297	118	<.001	32.950	7.282

**Caption:** Abbreviations: G-DYSLEXIA = Dyslexia group; G-CONTROL = Control group; M = mean; SD = standard deviation; F = *F* statistic (Levene’s test/ANOVA); Sig. = *p* value; η^2^ = eta squared (effect size); t = *t* statistic; df = degrees of freedom; Sig. (bilateral) = two-tailed *p*; Mean difference = difference in group means (G-DYSLEXIA − G-CONTROL); d = Cohen’s *d*.

**Table 2:** Results of differences in measures related to internalizing and externalizing emotional behavior between the G-DYSLEXIA and G-CONTROL groups, assessed using the SENA test

Behavioral and Emotional Problems	Groups		Levene's test for equality of variances			T test for equality of means				
	*G-DYSLEXIA*	*G-CONTROL*	*F*	*Sig.*	*η2*	*t*	*df*	*Sig. (bilateral)*	*Mean difference*	ð
**Internalizing Problems**										
*Depression*	M = 74.52 SD = 14.68	M = 30.63 SD = 10.81	8.935	0.003	0.847	18.636	118	<.001	43.883	12.898
*Anxiety*	M = 73.88 SD = 16.16	M = 28.35 SD = 9.29	32.239	<.001	0.748	18.875	118	<.001	45.533	13.213
*Social Anxiety*	M = 73.63 SD = 13.48	M = 27.82 SD = 9.42	9.780	0.002	0.884	21.575	118	<.001	45.817	11.632
*Somatic Complaints*	M = 71.12 SD = 14.13	M = 28.85 SD = 11.05	4.074	0.046	0.689	21.271	118	<.001	49.267	12.686
**Externalizing Problems**										
*Attention Problems*	M = 73.90 SD = 14.18	M = 28.68 SD = 9.69	9.680	0.002	0.588	20.388	118	<.001	45.217	12.148
*Hyperactivity-Impulsivity*	M = 74.72 SD = 14.01	M = 29.88 SD = 9.97	7.889	0.006	0.698	20.203	118	<.001	44.833	12.155
*Anger Control Problems*	M = 75.05 SD = 14.40	M = 30.87 SD = 10.88	7.520	0.007	0.612	18.961	118	<.001	44.183	12.763
*Aggression*	M = 71.28 SD = 14.51	M = 30.97 SD = 11.26	5.371	0.022	0.693	17.003	118	<.001	40.317	12.988
*Defiant Behavior*	M = 73.45 SD = 15.36	M = 29.18 SD = 10.38	18.934	<.001	0.788	18.486	118	<.001	44.267	13.116

**Caption:** Abbreviations: G-DYSLEXIA = Dyslexia group; G-CONTROL = Control group; M = mean; SD = standard deviation; F = *F* statistic (Levene’s test/ANOVA); Sig. = *p* value; η^2^ = eta squared (effect size); t = *t* statistic; df = degrees of freedom; Sig. (bilateral) = two-tailed *p*; Mean difference = difference in group means (G-DYSLEXIA − G-CONTROL); d = Cohen’s *d*.

**Table 3:** Results of Differences in Measures of Executive Functioning between G-DYSLEXIA and G-CONTROL, assessed using the ENFEN test

Executive Functions	Groups		Levene's test for equality of variances			T test for equality of means				
	*G-DYSLEXIA*	*G-CONTROL*	*F*	*Sig.*	*η2*	*t*	*df*	*Sig. (bilateral)*	*Mean Difference*	ð
*Resistance to Interference*	M = 24.22 SD = 6.77	M = 67.62 SD = 9.66	8.885	0.003	0.856	−28.494	118	<.001	−43.400	8.342
*Trails*	M = 13.68 SD = 5.75	M = 43.30 SD = 10.70	39.928	<.001	0.652	−18.879	118	<.001	−29.617	8.592
*Verbal Fluency*	M = 2.80 SD = 1.48	M = 11.87 SD = 2.01	4.190	0.043	0.796	−28.100	118	<.001	−9.067	1.767
*Rings*	M = 363.13 SD = 35.83	M = 147.80 SD = 23.89	19.471	<.001	0.751	38.727	118	<.001	215.333	30.455

**Caption:** Abbreviations: G-DYSLEXIA = dyslexia group; G-CONTROL = control group; M = mean; SD = standard deviation; F = *F* statistic (Levene’s test); Sig. = *p* value; η^2^ = eta squared (effect size); t = *t* statistic; df = degrees of freedom; Sig. (bilateral) = two-tailed *p*; Mean Difference = G-DYSLEXIA − G-CONTROL; d = Cohen’s *d*.

The results showed statistically significant differences between the G-DYSLEXIA and G-CONTROL groups across several key dimensions. In the behavioral profile, children with dyslexia scored significantly higher on scales such as Aggressiveness (M = 53.38, SD = 8.36) compared to the control group (M = 17.63, SD = 5.91), with t(118) = 27.022, *P* < 0.001, a large effect size (d = 7.246), and a substantial explained variance (*P* = 0.586). Similarly, significant differences were found in Anxiety (t(118) = 28.417, *P* < 0.001, d = 6.990, η^2^ = 0.689) and Depression (t(118) = 27.134, *P* < 0.001, d = 6.964, η^2^ = 0.521), where children with dyslexia showed higher levels compared to their peers without dyslexia.

Regarding emotional problems, children with dyslexia presented a higher prevalence of Depression (M = 74.52, SD = 14.68) and Social Anxiety (M = 73.63, SD = 13.48), with significant differences compared to the control group (*P* < 0.001 for both), reflected in t(118) = 18.636 (d = 12.898, η^2^ = 0.847) and t(118) = 21.575 (d = 11.632, η^2^ = 0.884), respectively. This suggests greater emotional vulnerability in the dyslexic group.

Finally, in executive functioning measures, children with dyslexia exhibited marked difficulties in Resistance to Interference (M = 24.22, SD = 6.77) compared to the control group (M = 67.62, SD = 9.66), with t(118) = −28.494, *P* < 0.001, d = 8.342, η^2^ = 0.856, and in Verbal Fluency (t(118) = −28.100, *P* < 0.001, d = 1.767, η^2^ = 0.796). These results highlight the considerable impact of dyslexia not only on academic skills but also on essential cognitive functions for learning and social adaptation.

Moreover, mediation analysis revealed that variables related to dyslexia diagnosis (internalizing emotional problems, executive functioning problems, and dyslexia diagnosis) had a significant effect on internalizing emotional problems [β = −41.41, *P* = 0.001], indicating a negative relationship between dyslexia diagnosis and the increase in internalizing emotional problems. Furthermore, executive functioning significantly affected emotional problems [β = −2.47, *P* = 0.01], suggesting that lower executive functioning is associated with increased internalization of emotional problems.

**Figure 1:**
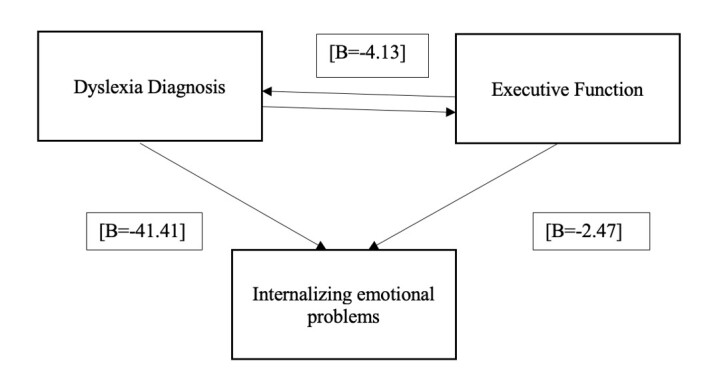
Mediation diagram between dyslexia, executive functioning, and internalizing symptoms

In turn, executive functioning showed a significant relationship with the diagnosis of dyslexia. When executive functioning was included as a mediating variable, the direct effect of dyslexia diagnosis on internalizing emotional problems decreased [β = −4.13, *P* = 0.001] but remained significant, indicating an indirect impact through executive functioning. This confirms that executive functioning partially mediates the relationship between dyslexia and internalizing symptoms (Figure 1).

A multiple linear regression model was performed to determine the dyslexia-related factors that best predicted the risk of developing internalizing emotional problems. In this analysis, the independent variables included dyslexia diagnosis and executive functioning. The results (Table 4) revealed that executive functioning is one of the strongest predictors of the development of internalizing emotional problems.

**Table 4:** Results of the multiple linear regression model predicting the risk of internalizing emotional problems

Variable	B	Standard Error	t	p-value	IC 95%
Dyslexia Diagnosis	−41.41	0.001	3.56	<.001	[-55.30, −30.22]
Executive Functioning	−2.47	0.01	4.89	<.001	[-3.52, −1.42]
Adjusted R^2^	0.755				

**Caption:** Abbreviations: B = unstandardized coefficient; Standard Error (SE) = standard error of B; t = *t* statistic; p-value (p) = significance level; 95% CI = 95% confidence interval for B; Adjusted R^2^ = coefficient of determination adjusted for the number of predictors.

The findings show that both dyslexia diagnosis and executive functioning are significant factors in predicting the development of internalizing emotional problems. The model indicates that 75.5% of the variability in depressive symptoms is explained by the variables analyzed.

Similarly, mediation analysis revealed that variables related to dyslexia diagnosis (executive functioning problems and dyslexia diagnosis) had a significant effect on externalizing emotional problems [β = −39.87, *P* = 0.001], indicating a negative relationship between dyslexia diagnosis and the increase in externalizing symptoms. Moreover, executive functioning also significantly affected externalizing emotional problems [β = −3.05, *P* = 0.01], suggesting that lower executive functioning is associated with an increase in externalizing symptoms.

**Figure 2:**
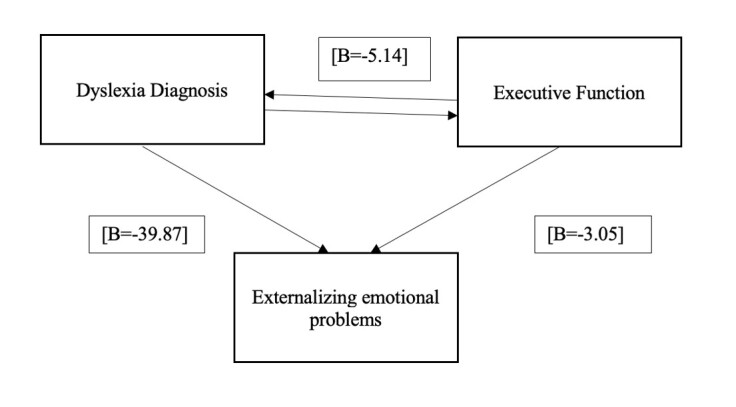
Mediation diagram between dyslexia, executive functioning, and externalizing emotional problems

In turn, executive functioning showed a significant relationship with the diagnosis of dyslexia. When executive functioning was included as a mediating variable, the direct effect of dyslexia diagnosis on externalizing emotional problems decreased [β = −5.14, *P* = 0.001] but remained significant, indicating an indirect impact through executive functioning. This confirms that executive functioning partially mediates the relationship between dyslexia and externalizing emotional problems (Figure 2).

A multiple linear regression model was performed to determine the dyslexia-related factors that best predict the risk of developing externalizing emotional problems. In this analysis, the independent variables included dyslexia diagnosis and executive functioning. The results (Table 5) revealed that executive functioning is one of the strongest predictors of the development of externalizing emotional problems.

**Table 5:** Results of the multiple linear regression model predicting the risk of externalizing emotional problems

Variable	B	Standard Error	t	p-value	IC 95%
Dyslexia Diagnosis	−39.87	0.001	3.56	<.001	[-52.80, −28.94]
Executive Functioning	−3.05	0.01	4.89	<.001	[-4.32, −1.78]
Adjusted R^2^	0.641				

**Caption:** Abbreviations: B = unstandardized coefficient; Standard Error (SE) = standard error of B; t = *t* statistic; p-value (p) = significance level; 95% CI = 95% confidence interval for B; Adjusted R^2^ = coefficient of determination adjusted for the number of predictors.

The findings show that both dyslexia diagnosis and executive functioning are significant factors in predicting the development of externalizing emotional problems. The model indicated that 64.1% of the variability in externalizing emotional problems is explained by the variables included in the model.

## Discussion

The present study aimed to explore and compare the behavioral, emotional, and executive functioning profiles between children with dyslexia and those with typical reading development. Dyslexia, a specific learning disorder primarily affecting reading, has traditionally been associated with academic difficulties; however, recent studies have shown that its impact is much broader, affecting the emotional well being, behavior, and executive functions of children with dyslexia^(44,45)^.

One of the most relevant findings of our study was the identification of a higher prevalence of emotional problems, specifically anxiety and depression, in children with dyslexia than in those without this disorder^(46,47)^. This result aligns with previous research indicating that children with dyslexia are at greater risk of developing emotional disorders, such as anxiety and depression^(48)^, due to repeated experiences of academic failure and social stigmatization^(49)^. For example, a systematic review by Wilson et al.^(50)^ highlighted that children with dyslexia are significantly more likely to experience depressive symptoms, which are associated with their inability to meet academic and social expectations. This situation can lead to low self-esteem and hopelessness, further reinforcing feelings of anxiety and depression. Moreover, these emotional problems can create a cycle in which anxiety and depression intensify learning difficulties, adding another layer of limitation to academic progress^(49)^.

It is also important to consider that dyslexia not only affects academic skills but also has a profound impact on children’s mental health^(46)^. Academic pressure and constant comparison with peers without learning difficulties can increase stress levels, negatively affecting the emotional well being of these children^(47,48)^. Therefore, early intervention and psychological support have emerged as fundamental strategies to mitigate these effects and improve the quality of life of children with dyslexia. Providing an inclusive learning environment that is sensitive to emotional needs can significantly reduce levels of anxiety and depression, thereby fostering a more balanced development in this clinical population^(50)^.

Our study’s results also indicated that children with dyslexia exhibit a greater degree of behavioral problems, such as hyperactivity, impulsivity, and defiant behaviors, compared to their peers without dyslexia. This finding is consistent with literature suggesting that learning difficulties, such as dyslexia, may be associated with increased disruptive behaviors, possibly as a response to frustration and stress linked to low academic performance^(51,52)^. It is important to consider that the behavioral problems observed in children with dyslexia may reflect their internal struggle to adapt to academic demands they cannot meet due to their reading difficulties. These behaviors may be interpreted as attempts to cope with or express accumulated frustration, resulting in defiant or disruptive behaviors in the classroom^(53)^.

Additionally, the lack of understanding from teachers and peers can exacerbate these behaviors, creating a hostile environment for the child, which may lead to social isolation and increased problematic behaviors^(54)^. An interesting point to consider is that while behavioral problems may be more visible and therefore easier to identify, their underlying origin in dyslexia may be more complex. Difficulties in emotional self-regulation and impulse control—characteristics observed in children with dyslexia—appear to be linked to deficits in executive functions, impairing their ability to adequately manage emotions^(55)^.

Executive functioning encompasses a set of cognitive processes that are crucial for adapting to academic and social demands, including working memory, cognitive flexibility, planning, and inhibition [24â€“26]. Our study revealed that children with dyslexia present significant alterations in these functions compared to their peers without dyslexia, which is consistent with previous research highlighting executive function difficulties as a key component of dyslexia^(56,57)^. The difficulties in executive functions observed in the dyslexia group may partly explain why these children struggle to organize and plan school tasks, maintain attention over long periods, and adapt to task changes^(51)^.

These skills are essential not only for academic performance but also for daily life, as they enable individuals to manage multiple tasks, make informed decisions, and regulate emotions^(58)^. Moreover, neuropsychological studies have suggested that executive function difficulties in children with dyslexia may be linked to alterations in the brain networks supporting these functions^(18-22)^, particularly in areas such as the prefrontal cortex^(59)^. Such alterations could be one of the reasons why children with dyslexia perform worse in tasks requiring higher cognitive control, such as planning and inhibition^(60)^.

These findings have significant implications for educational interventions. Programs that include executive function training, such as improving working memory or planning skills, could help children with dyslexia overcome cognitive barriers. Furthermore, incorporating teaching strategies tailored to these children’s cognitive strengths and weaknesses could improve not only their academic performance but also their self-esteem and overall well being^(59-61)^.

It is important to note that many of the emotional symptoms observed in children with dyslexia may be interpreted as indirect consequences of persistent reading difficulties. The frustration associated with lower academic performance, feelings of incompetence compared to peers, and pressure to meet academic goals not adapted to their needs contribute to the development of anxiety, sadness, demotivation, and low self-esteem. In this sense, dyslexia not only represents a cognitive challenge but also a condition that can trigger a process of progressive emotional vulnerability if its secondary effects in educational and social environments are not properly addressed.

In line with these observations, the Risk and Resilience model proposed by Hoeft et al.^(62)^ provides a valuable conceptual framework for understanding the complexity of dyslexia beyond the academic domain. This model suggests that while reading difficulties pose a significant risk of emotional development, individual and contextual protective factors can modulate their negative effects. Self-esteem, family support, coping strategies, and brain plasticity are among these factors. Neuroimaging studies, such as those conducted by Hoeft et al.^(62)^, have shown that some children with dyslexia achieve functional adaptation through neural compensation networks, highlighting the importance of fostering positive and resilient learning environments. Incorporating this approach not only helps explain why some children with dyslexia show fewer emotional symptoms than others but also informs interventions that strengthen these protective capacities.

Finally, another key finding of this study was the identification of executive functioning as a predictor of both internalizing and externalizing emotional problems in children with dyslexia. From an educational perspective, these findings reinforce the need to design intervention programs that integrate executive function training into the school curriculum. Strategies such as using graphic organizers, visual routines, emotional self-regulation techniques, and structured games can facilitate planning, inhibition, and cognitive flexibility in children with dyslexia.

Moreover, implementing methodological adaptations, such as breaking down complex tasks into smaller steps, providing extra time for assignments, or using visual aids, can contribute to a more accessible learning environment. Training teachers to recognize the specific difficulties of these children and to use neuroeducation-based pedagogical tools can reduce students’ frustration and foster active participation, improving both academic performance and emotional well being in the classroom.

The results showed that dyslexia diagnosis has a significant negative effect on internalizing emotional problems. These findings are consistent with those of previous studies demonstrating that children with dyslexia are at greater risk of developing emotional symptoms due to frustration and persistent academic difficulties^(48,63)^. Research by Snowling and Hulme^(64)^ highlighted that dyslexia not only affects reading skills but also has a significant impact on children’s emotional well being, intensified by constant experiences of academic failure.

The mediating role of executive functioning in this relationship is particularly relevant. This study showed that executive functioning has a direct effect on emotional problems, both internalizing and externalizing, suggesting that deficits in these cognitive functions exacerbate emotional symptoms in children with dyslexia. These findings align with previous research showing that executive functioning is fundamental for emotional regulation and impulse control, which in turn may influence the emergence of internalizing symptoms such as anxiety and depression^(65)^.

Indeed, Diamond^(66)^ argued that children with executive function difficulties, such as those with dyslexia, are more prone to developing emotional disorders due to their inability to manage academic stress and social demands. The reduction of the direct effect of dyslexia on emotional problems after including executive functioning as a mediating variable reinforces the importance of this factor in the relationship between dyslexia and mental health.

This finding underscores the need for interventions that focus not only on the academic skills of children with dyslexia but also on strengthening their executive functioning skills as a way to mitigate the emotional impact associated with this disorder^(67)^. Furthermore, the association between dyslexia and externalizing emotional problems, such as aggression and defiant behavior, has been widely documented in the literature. This study confirms this relationship, showing a significant effect of dyslexia diagnosis on externalizing symptoms, consistent with research indicating that children with learning difficulties are more likely to display disruptive behaviors as a way of coping with frustration and anxiety^(68,69)^.

Despite the important findings of this study, some limitations should be noted. One major limitation is the reliance on self-reports and parent-reported assessments, which may introduce bias in evaluating emotional and behavioral problems. Future studies could benefit from incorporating more objective assessments, such as direct classroom observations or neuropsychological tests administered by professionals, to provide a more comprehensive and accurate evaluation.

Similarly, although this study focused on comparisons between children with and without dyslexia, future research could explore how additional factors, such as family environment, quality of school support, and previous interventions, influence behavioral, emotional, and executive functioning profiles in children with dyslexia. It would also be important to investigate the effectiveness of different intervention programs in improving these profiles and reducing gaps between children with and without dyslexia.

Finally, continued research on the neurobiological underpinnings of dyslexia and how they affect the development of executive and emotional functions is necessary. A better understanding of these relationships could lead to more personalized and effective interventions that not only address dyslexia symptoms but also improve the overall well being and quality of life of children with this diagnosis.

This study makes an original contribution by demonstrating the mediating role of executive functioning in the relationship between dyslexia and emotional symptoms, an area that has been scarcely explored in Spanish-speaking child populations. By integrating behavioral, emotional, and cognitive measures, this work provides a comprehensive view of the multidimensional impact of dyslexia. Identifying specific patterns among these variables not only enriches the theoretical understanding of the disorder but also opens new research and intervention pathways to address the needs of children with dyslexia in both school and clinical settings.

## Conclusions

In conclusion, this study highlights that children with dyslexia exhibit significant differences in emotional, behavioral, and executive functioning profiles compared to their peers without dyslexia. The results indicate that children with dyslexia are more likely to experience disorders such as anxiety, depression, or behavioral problems, including hyperactivity and impulsivity, which are associated with deficits in executive functioning such as inhibition, planning, and working memory. These difficulties not only affect their academic performance but also have a considerable impact on their social adaptation and overall well being. These findings underscore the importance of addressing dyslexia not only from an academic perspective but also by considering its emotional and behavioral implications, emphasizing the need for an interdisciplinary approach to the study and understanding of this learning disorder.
