# Supplementary material for: Executive, behavioural and emotional functioning in Spanish children with dyslexia
Source: Codas. 2025 Dec 12;37(6):e20240352. doi: 10.1590/2317-1782/e20240352en (PMC12704611; doi:10.1590/2317-1782/e20240352en)
Supplement: Translation

# Translation: Funcionamiento ejecutivo, conductual y emocional en niños españoles con dislexia

**DOI:** 10.1590/2317-1782/e20240352es

**Authors:** Miguel López-Zamora, Nadia Porcar-Gozalbo, Alejandro Cano-Villagrasa, Isabel López-Chicheri

**Affiliations:** 1 Departamento de Psicología Evolutiva y de la Educación, Facultad de Psicología y Logopedia, Universidad de Málaga – UMA - Málaga (Andalucia), España.; 2 Facultad de Ciencias de la Salud, Universidad Internacional de Valencia – VIU - Valencia (Comunidad Valenciana), España.; 3 Universidad Católica de Murcia – UCAM - Murcia (Región de Murcia), España.

**Keywords:** **es:** Dislexia, Funciones Ejecutivas, Ansiedad, Depresión, Trastornos del Comportamiento

## Abstract

**Objetivo:**

La dislexia es un trastorno específico del aprendizaje que afecta la lectura y se asocia con problemas emocionales y conductuales. Investigaciones previas indican que los niños con dislexia tienen mayor riesgo de desarrollar ansiedad, depresión y dificultades en las funciones ejecutivas, lo que afecta su rendimiento académico y bienestar.

**Método:**

Este estudio exploró y comparó los perfiles conductuales, emocionales y de funcionamiento ejecutivo en niños con dislexia y en niños normotípicos en una muestra de 120 niños de 8 a 10 años, divididos en un grupo con dislexia y un grupo control. Se usaron el BASC-3, SENA y ENFEN para las evaluaciones, y los datos se analizaron mediante pruebas t, el test de Levene y análisis de mediación.

**Resultados:**

Los niños con dislexia mostraron significativamente mayores niveles de agresividad, ansiedad, depresión y problemas de atención. Además, presentaron dificultades en funciones ejecutivas como resistencia a la interferencia y fluidez verbal, evidenciando el impacto de la dislexia en estas áreas.

**Conclusión:**

Los análisis de mediación sugieren que la dislexia es un indicador potencial de dificultades en las funciones ejecutivas y la conducta, además de influir en problemas emocionales interiorizados y exteriorizados. Estos hallazgos destacan la necesidad de implementar estrategias educativas y terapéuticas integrales para atender las necesidades de esta población vulnerable.

## INTRODUCCIÓN

El desarrollo de los procesos específicos de aprendizaje de la lectura representa un desafío significativo para la trayectoria madurativa y académica de los niños, constituyendo un factor determinante en el éxito educativo durante las primeras etapas escolares^(1,2)^. La lectura, a diferencia del lenguaje, es un proceso neurocognitivo relativamente reciente que no cuenta con una base anatómica predeterminada en el cerebro, lo que explica la necesidad de una enseñanza explícita para su adquisición^(3-5)^. Este aprendizaje requiere una organización precisa del sistema cortical, que integre de manera eficaz los diversos procesos implicados en la lectura. Sin embargo, diversos factores pueden generar conexiones corticales ineficaces, lo que provoca dificultades en la adquisición y el desarrollo de la lectura^(6-8)^.

Entre los trastornos que pueden afectar a la adquisición de la lectura, la dislexia es uno de los más relevantes. Se trata de un trastorno específico del aprendizaje que afecta a entre el 4.66% y el 9.22% de los niños en edad escolar en Europa^(9,10)^. A nivel global, una revisión sistemática realizada por Yang et al.^(11)^, que incluyó 58 estudios publicados desde la década de 1950 hasta 2021, reveló una prevalencia combinada del 7.1%. Esta revisión, que abarcó escolares de entre 6 y 13 años en 16 países, incluidos seis países de ingresos bajos, mostró estimaciones de prevalencia similares en los países de ingresos altos, situándose entre el 6.8% y el 8.3%. Estos datos subrayan la importancia de estudiar y comprender las bases neurocognitivas del aprendizaje de la lectura, así como los factores que influyen en su desarrollo, para abordar de manera adecuada los efectos de la dislexia.

Sin embargo, a pesar del avance en la caracterización de los perfiles neurocognitivos asociados a la dislexia, persiste una laguna importante en la literatura en relación con la comprensión integrada de los factores emocionales, conductuales y ejecutivos implicados^(7,8)^. La mayoría de los estudios tienden a abordar estas dimensiones de manera aislada, sin explorar suficientemente las posibles relaciones de mediación entre ellas^(10,11)^. En particular, el rol de las funciones ejecutivas como posibles mecanismos explicativos que vinculen las dificultades lectoras con los síntomas emocionales y conductuales permanece subexplorado, lo que limita tanto la interpretación de los hallazgos como el desarrollo de intervenciones más específicas y eficaces^(11)^.

Los niños con dislexia presentan dificultades en el reconocimiento de palabras, la ortografía y la decodificación, a pesar de tener una inteligencia normotípica y no presentar déficits neurológicos o sensoriales que dificulten esta competencia^(12,13)^. Es por ello por lo que el proceso de aprendizaje de la lectura supone para la población infantil con dislexia una limitación significativa, constante y frustrante, que tiene un impacto negativo en su vida^(14)^.

Investigaciones recientes han reportado que casi el 60% de los niños con un diagnóstico principal de dislexia cumplen con los criterios para al menos un trastorno relacionado con el estado del ánimo, ansiedad o depresión^(15-20)^, y que además existe una alta proporción de trastornos de ansiedad y depresión entre los niños con dislexia. Burke et al.^(21)^ han mostrado que las personas con dificultades en los procesos lectores presentan tasas significativamente más altas de trastornos de internalización y externalización que las personas sin una alteración en la lectura. En la misma línea, Wang^(22)^ mostró que los niños con dislexia que tienen niveles más altos de ansiedad y depresión muestran un rendimiento más bajo en sus habilidades académicas y de aprendizaje

Para la población infantil con dislexia, un menor rendimiento académico predice un aumento de los síntomas de ansiedad y depresión^(23)^. Los estudios han identificado que el estrés y la ansiedad relacionados con la escuela son más prevalentes en los estudiantes de educación primaria con dislexia^(24)^ en comparación con estudiantes de niveles académicos más elevados, como por ejemplo los universitarios^(25)^. Debido a que los niños con dislexia se autoperciben a sí mismos como peores en las tareas académicas, suelen desarrollar conductas disruptivas y de aislamiento social en las aulas^(26)^, reduciendo su autoestima y distorsionando su autoconcepto^(27)^. Para autores como Alexander-Passe^(28)^, estas conductas disruptivas están provocadas por la ansiedad, que a su vez es producida por los sentimientos ansiosos, lo que cristaliza en que los niños con dislexia suelan interrumpir en clase, no prestar atención a las explicaciones del profesor, o se peleen con sus compañeros.

De igual forma, la baja autoestima puede ser una causa de depresión en los niños con dislexia^(20,29)^. Sin embargo, los resultados en la literatura son inconsistentes. Los síntomas internalizantes se refieren a dificultades emocionales que se manifiestan internamente, como la ansiedad, la depresión, el aislamiento social o la baja autoestima. Estas problemáticas suelen ser menos visibles para los demás, pero afectan profundamente al bienestar emocional de la persona. Por otro lado, los síntomas externalizantes implican comportamientos que se manifiestan hacia el exterior, como la agresividad, la impulsividad o la conducta disruptiva, siendo más notables por su impacto en el entorno. En este contexto, los estudios de Katsantonis et al.^(30)^ y Miller et al.^(31)^ no han encontrado una relación significativa entre la dislexia y niveles elevados de síntomas de internalización, como ansiedad o depresión, sugiriendo que las dificultades emocionales que experimentan estos niños pueden no ser tan evidentes en términos de síntomas internalizados. Por ende, no existe un consenso global que permita describir con claridad los resultados de la relación entre el acoso escolar, la victimización y la aparición de trastornos de ansiedad y del estado del ánimo.

Además, aparte de las evidencias sobre las alteraciones emocionales de las personas con dificultades específicas del aprendizaje de la lectura, se ha encontrado que el perfil específico de funcionamiento ejecutivo en esta población puede tener una estrecha relación con los trastornos de ansiedad, de depresión y de conducta^(32)^. La población infantil con diagnóstico de dislexia presenta déficit en el funcionamiento ejecutivo^(33-35)^, independientemente de las dificultades adicionales que puedan aparecer o establecerse de forma comórbida^(36,37)^. Existe evidencia empírica consistente de que los niños pequeños con dislexia exhiben un bajo rendimiento en tareas de funciones ejecutivas que involucran procesos motivacionales y emocionales^(38)^.

También se encuentra un deterioro en funciones como la inhibición conductual, la cual están asociadas con comportamiento agresivo y trastorno de conducta en niños de primaria (6-12 años) y preescolares (4 años)^(39)^, en comparación con otras funciones como la planificación y la memoria de trabajo. Sin embargo, la literatura científica no arroja un patrón común en las alteraciones en el funcionamiento ejecutivo presentadas por los niños con dislexia. Esto es debido, en gran parte, a la heterogeneidad de los participantes y de las diferencias individuales de los mismos, lo que causa una compleja situación para comparar los resultados entre los estudios publicados hasta la fecha.

En este sentido, una mayor comprensión del funcionamiento ejecutivo y su papel en la dislexia no solo tiene implicaciones teóricas, sino también un alto valor aplicado. Identificar perfiles específicos de alteración ejecutiva podría permitir el diseño de estrategias de intervención más personalizadas, que aborden no solo las dificultades lectoras, sino también los síntomas emocionales y conductuales asociados. Esto resulta especialmente relevante para los profesionales de la salud y la educación, ya que podría facilitar la implementación de programas preventivos y terapéuticos en contextos escolares, promoviendo un enfoque interdisciplinar y centrado en las necesidades integrales del alumnado con dislexia.

Por todo ello, el objetivo principal del presente estudio fue explorar las diferencias en los procesos conductuales, el patrón de conductas emocionales internalizantes y externalizantes, así como el funcionamiento ejecutivo en una cohorte de niños con dislexia, comparando los resultados de las mediciones en cada uno de los apartados con otro grupo de menores con un desarrollo normotípico. De la misma manera, se exploró la relación entre todas estas variables y cómo fue su efecto en la mediación entre ellas.

## MÉTODO

### Participantes

Se seleccionó un total de 120 participantes (58 niñas y 62 niños) con edades comprendidas entre 8 y 10 años (M=9.2). Los participantes diagnosticados con dislexia habían sido evaluados por su hospital de referencia y centro educativo, siguiendo los criterios diagnósticos establecidos en el DSM-5 TR^(9)^. La herramienta de evaluación utilizada por los profesionales de los centros de salud o educativos (psicólogos y logopedas) para diagnosticar la dislexia fue el test PROLEC-R^(40)^. La muestra se dividió en dos grupos: un grupo experimental compuesto por 60 individuos diagnosticados con dislexia (G-DISLEXIA), y un grupo de comparación formado por 60 individuos con un desarrollo típico de la lectura (G-CONTROL). Todos los participantes del estudio eran hablantes monolingües de español nacidos en España.

Se aplicaron una serie de criterios de inclusión y exclusión para formar los grupos de la muestra. Los criterios de inclusión para el grupo G-DISLEXIA fueron los siguientes: tener un diagnóstico de dislexia, estar entre los 8 y 10 años de edad, y poseer un lenguaje expresivo. Por otro lado, los criterios de exclusión para los participantes en ambos grupos (G-DISLEXIA y G-CONTROL) incluyeron tener una patología sensorial grave, un diagnóstico de discapacidad intelectual, o sospecha de trastorno psiquiátrico grave u otra condición preexistente que dificulte la evaluación.

### Instrumentos y materiales

#### BASC-3, Sistema de Evaluación de la Conducta de Niños y Adolescentes – 3

El BASC-3, Sistema de Evaluación de la Conducta de Niños y Adolescentes - 3 (BASC-3)^(41)^ es una herramienta clínica utilizada para evaluar las emociones y conductas de niños y adolescentes, detectando trastornos desadaptativos en contextos como la familia y la escuela. El BASC-3 incluye un autoinforme para el paciente y dos cuestionarios dirigidos a padres (P) y a profesores o tutores (T). Para los fines de esta investigación, se considerarán únicamente los cuestionarios dirigidos a padres y profesores. El alfa de Cronbach de esta prueba es de 0.90.

#### Cuestionario SENA, Sistema de Evaluación de Niños y Adolescentes

El Cuestionario SENA, Sistema de Evaluación de Niños y Adolescentes (SENA)^(42)^ se compone de un conjunto de 9 cuestionarios diseñados para tres grupos de edad específicos: Infantil (3-6 años), Primaria (6-12 años) y Secundaria (12-18 años). Este sistema incluye cuestionarios específicos para recoger información de diferentes informadores en los principales contextos en los que el niño se desenvuelve, como la familia y la escuela. Además, incorpora tres modelos de autoinforme adaptados a la edad del evaluado, aplicables a partir de los 6 años. En cada cuestionario, los informadores deben calificar la frecuencia con la que aparece la conducta descrita utilizando una escala de cinco puntos (desde Nunca o casi nunca hasta Siempre o casi siempre), excepto en el autoinforme para los niños de 6-8 años, que utiliza una escala de tres opciones: Sí, No y A veces. El alfa de Cronbach de esta prueba es de 0.81.

#### ENFEN, Evaluación Neuropsicológica de las Funciones Ejecutivas en Niños

El ENFEN, Evaluación Neuropsicológica de las Funciones Ejecutivas en Niños (ENFEN)^(43)^ es una herramienta diseñada para evaluar de manera individual el nivel de madurez y el rendimiento cognitivo en tareas relacionadas con las funciones ejecutivas en niños de 6 a 12 años. La batería consta de cuatro pruebas (Fluidez verbal, Construcción de senderos, Construcción con anillas y Resistencia a la interferencia) que miden diferentes aspectos de las funciones ejecutivas. Los resultados obtenidos permiten un diagnóstico más profundo y orientan la intervención neuropsicológica, tanto en niños sanos como en aquellos que presentan retrasos en el desarrollo o alteraciones cognitivas o emocionales derivadas de disfunciones o daños cerebrales. El alfa de Cronbach de esta prueba es de 0.84.

### Procedimiento

El estudio fue aprobado por el comité de ética de la Universidad de Málaga (UMA). Todas las familias de los participantes aceptaron formar parte del estudio mediante la firma de un consentimiento informado, garantizando el anonimato y la protección de datos. La recolección de datos se realizó en dos etapas. En la primera, se llevó a cabo una entrevista inicial y la administración de pruebas de evaluación de la conducta, la emoción y el funcionamiento ejecutivo. Se realizaron dos sesiones individuales, cada una con una duración aproximada de 45 minutos, espaciadas entre 3 y 5 días hábiles, según la disponibilidad del centro educativo y las necesidades del menor. Este intervalo permitió evitar la fatiga y asegurar una evaluación más precisa y cómoda para los participantes. La aplicación de las pruebas fue realizada por un equipo de profesionales entrenados compuesto por psicólogos educativos y neuropsicólogos con experiencia en evaluación infantil. Todos los evaluadores recibieron formación específica para la administración estandarizada de cada instrumento, con el fin de garantizar la fiabilidad de los datos obtenidos. En la segunda etapa, se realizó un análisis de los datos registrados de los participantes del estudio, utilizando las estadísticas seleccionadas para esta investigación y generando una base de datos con los resultados de los individuos evaluados.

### Diseño

Este es un estudio descriptivo y transversal que cuenta con un grupo experimental (G-DISLEXIA) y un grupo control (G-CONTROL). La elección de un diseño transversal, en lugar de longitudinal, responde a la necesidad de evaluar diferencias significativas en un momento puntual del desarrollo escolar temprano, etapa en la que se manifiestan de forma más clara las dificultades lectoras y sus posibles repercusiones emocionales y conductuales. Este tipo de diseño permite comparar de forma eficaz dos grupos bien definidos dentro de un rango de edad homogéneo, facilitando una interpretación más directa de los resultados en relación con el diagnóstico de dislexia. Las variables dependientes fueron las funciones y habilidades evaluadas por los instrumentos estandarizados mencionados anteriormente. La variable independiente del estudio fue el diagnóstico de dislexia en esta población. Para analizar las variables del estudio, primero se realizó una prueba de normalidad utilizando el test de Chi-cuadrado para las variables sociodemográficas de los participantes, como *Sexo, Edad, Diagnóstico, Años de tratamiento, Comorbilidad, Apoyo escolar, Semanas de gestación, y Apgar*. Para examinar la relación y las tendencias entre las variables relativas a los componentes relativos al perfil conductual, emocional y de funcionamiento ejecutivo se llevó a cabo un análisis estadístico descriptivo junto con una prueba t para muestras dependientes con el fin de observar diferencias entre los dos grupos. Igualmente, para observar la relación y la influencia entre las variables del presente estudio, se empleó un análisis de mediación entre las variables relativas a los problemas emocionales y la dislexia. Todos estos análisis se llevaron a cabo a través del software SPSS en su versión 29.

## RESULTADOS

Se llevó a cabo un análisis descriptivo sobre las variables sociodemográficas de sexo, edad, diagnóstico de dislexia, años de tratamiento, comorbilidad, apoyo escolar, semanas de gestación y puntaje de Apgar, junto con los resultados de la prueba de Chi-cuadrado para cada variable. Los resultados no indicaron diferencias significativas en ninguna de las variables (p>0.05).

Para dar respuesta a los objetivos planteados en el presente estudio, se reflejan a continuación los resultados obtenidos en los análisis de prueba T realizados para la comparación de las puntuaciones de los dos grupos en las medidas relativas a la conducta, la conducta emocional interiorizada y exteriorizada, así como en el funcionamiento ejecutivo. La diferencia en las puntuaciones se calculó utilizando la prueba T para muestras independientes en relación con las variables de competencias cognitivas. Los resultados revelaron diferencias significativas en todas las variables relacionadas con el funcionamiento ejecutivo. Posteriormente, se realizó la prueba de Levene para cada variable, donde se encontró que se cumplía el supuesto de homogeneidad de varianzas, ya que el valor de significancia superó 0.05 (p > 0.05), lo que indica que las varianzas de los grupos estudiados en las variables relativas a las medidas evaluadas son homogéneas (Tabla 1, 2 y 3).

**Tabla 1:** Resultados de las diferencias en las medidas relativas al perfil conductual entre el G-DYSLEXIA y G-CONTROL, evaluadas a través del BASC-3

Perfil Conductual	Grupos		Levene's test for equality of variances			T test for equality of means				
	*G-DYSLEXIA*	*G-CONTROL*	*F*	*Sig.*	*η^2^*	*t*	*gl*	*Sig. (bilateral)*	*Diferencia de medas*	ð
**Puntuaciones de las escalas compuestas, clínicas y adaptativas**										
*Agresividad*	M = 53.38 DT = 8.36	M = 17.63 DT = 5.91	14.878	<.001	.586	27.022	118	<.001	35.750	7.246
*Ansiedad*	M = 55.63 DT = 7.44	M = 19.37 DT = 6.49	1.254	.265	.689	28.417	118	<.001	36.267	6.990
*Depresión*	M = 54.13 DT = 7.34	M = 19.63 DT = 6.56	.698	.405	.521	27.134	118	<.001	34.500	6.964
*Somatización*	M = 52.28 DT = 7.70	M = 18.77 DT = 6.22	2.227	.138	.855	26.210	118	<.001	33.517	7.049
*Atipicidad*	M = 54.95 DT = 8.10	M = 18.85 DT = 6.05	11.679	<.001	.971	27.631	118	<.001	36.100	7.156
*Retraimiento*	M = 53.48 DT = 8.06	M = 19.93 DT = 5.85	10.826	.001	.685	26.066	118	<.001	33.550	7.050
*Problemas de atención*	M = 53.28 DT = 7.61	M = 20.13 DT = 5.40	10.830	.001	.753	27.497	118	<.001	33.150	6.603
*Adaptabilidad*	M = 55.08 DT = 8.12	M = 19.38 DT = 6.24	5.360	.022	.698	26.978	118	<.001	35.700	7.248
*Habilidades sociales*	M = 55.33 DT = 8.54	M = 19.83 DT = 5.43	20.250	<.001	.611	27.160	118	<.001	35.500	7.159
*Actividades cotidianas*	M = 53.53 DT = 7.39	M = 19.57 DT = 5.59	3.038	.084	.684	28.361	118	<.001	33.967	6.560
*Comunicación funcional*	M = 54.45 DT = 8.23	M = 18.20 DT = 5.99	5.155	.025	.752	26.227	118	<.001	35.500	7.414
**Puntuaciones de las escalas de contenido**										
*Control de la ira*	M = 54.48 DT = 8.49	M = 18.20 DT = 5.99	8.091	.005	.814	27.039	118	<.001	36.283	7.350
*Acoso escolar*	M = 53.55 DT = 7.05	M = 19.30 DT = 6.20	.472	.493	.862	28.239	118	<.001	34.250	6.643
*Trastornos del desarrollo social*	M = 56.15 DT = 7.66	M = 19.42 DT = 5.92	5.693	.019	.834	29.368	118	<.001	36.733	6.851
*Autocontrol emocional*	M = 55.00 DT = 7.72	M = 19.32 DT = 5.98	7.390	.008	.812	28.293	118	<.001	35.683	6.908
*Funcionamiento ejecutivo*	M = 53.57 DT = 7.72	M = 17.73 DT = 6.46	3.045	.084	.877	27.560	118	<.001	35.833	7.122
*Emocionalidad negativa*	M = 55.88 DT = 7.45	M = 18.43 DT = 5.51	6.806	.010	.683	27.560	118	<.001	37.450	6.554
*Resiliencia*	M = 52.18 DT = 8.44	M = 19.23 DT = 5.89	6.490	.012	.509	31.297	118	<.001	32.950	7.282

**Subtítulo:** Abreviaturas: G-DYSLEXIA = Grupo con dislexia; G-CONTROL = Grupo control; M = Media; DT = Desviación típica; F = Estadístico F (prueba de Levene/ANOVA); Sig. = Nivel de significación (*p*); η^2^ = Eta cuadrado (tamaño del efecto); t = Estadístico *t*; gl = Grados de libertad; Sig. (bilateral) = *p* de dos colas; Diferencia de medias = Diferencia entre las medias (G-DYSLEXIA − G-CONTROL); d = Tamaño del efecto de Cohen.

**Tabla 2:** Resultados de las diferencias en las medidas relativas a la conducta emocional interiorizada y exteriorizada entre el G-DYSLEXIA y G-CONTROL, evaluadas a través de la prueba SENA

Problemas conductuales y emocionales	Grupos		Levene's test for equality of variances			T test for equality of means				
	*G-DYSLEXIA*	*G-CONTROL*	*F*	*Sig.*	*η^2^*	*t*	*gl*	*Sig. (bilateral)*	*Diferencia de medias*	ð
**Problemas interiorizados**										
*Depresión*	M = 74.52 DT = 14.68	M = 30.63 DT = 10.81	8.935	.003	.847	18.636	118	<.001	43.883	12.898
*Ansiedad*	M = 73.88 DT = 16.16	M = 28.35 DT = 9.29	32.239	<.001	.748	18.875	118	<.001	45.533	13.213
*Ansiedad social*	M = 73.63 DT = 13.48	M = 27.82 DT = 9.42	9.780	.002	.884	21.575	118	<.001	45.817	11.632
*Quejas somáticas*	M = 71.12 DT = 14.13	M = 28.85 DT = 11.05	4.074	.046	.689	21.271	118	<.001	49.267	12.686
**Problemas exteriorizados**										
*Problemas de atención*	M = 73.90 DT = 14.18	M = 28.68 DT = 9.69	9.680	.002	.588	20.388	118	<.001	45.217	12.148
*Hiperactividad-impulsividad*	M = 74.72 DT = 14.01	M = 29.88 DT = 9.97	7.889	.006	.698	20.203	118	<.001	44.833	12.155
*Problemas de control de la ira*	M = 75.05 DT = 14.40	M = 30.87 DT = 10.88	7.520	.007	.612	18.961	118	<.001	44.183	12.763
*Agresión*	M = 71.28 DT = 14.51	M = 30.97 DT = 11.26	5.371	.022	.693	17.003	118	<.001	40.317	12.988
*Conducta desafiante*	M = 73.45 DT = 15.36	M = 29.18 DT = 10.38	18.934	<.001	.788	18.486	118	<.001	44.267	13.116

**Subtítulo:** Abreviaturas: G-DYSLEXIA = Grupo con dislexia; G-CONTROL = Grupo control; M = Media; DT = Desviación típica; F = Estadístico F (prueba de Levene/ANOVA); Sig. = Nivel de significación (*p*); η^2^ = Eta cuadrado (tamaño del efecto); t = Estadístico *t*; gl = Grados de libertad; Sig. (bilateral) = *p* de dos colas; Diferencia de medias = Diferencia entre las medias (G-DYSLEXIA − G-CONTROL); d = Tamaño del efecto de Cohen.

**Tabla 3:** Resultados de las diferencias en las medidas relativas al funcionamiento ejecutivo entre G-DYSLEXIA y G-CONTROL a través de la prueba ENFEN

Funciones ejecutivas	Grupos		Levene's test for equality of variances			T test for equality of means				
	*G-DYSLEXIA*	*G-CONTROL*	*F*	*Sig.*	*η^2^*	*t*	*gl*	*Sig. (bilateral)*	*Diferencia de medias*	ð
*Resistencia a la interferencia*	M = 24.22 DT = 6.77	M = 67.62 DT = 9.66	8.885	.003	.856	-28.494	118	<.001	-43.400	8.342
*Senderos*	M = 13.68 DT = 5.75	M = 43.30 DT = 10.70	39.928	<.001	.652	-18.879	118	<.001	-29.617	8.592
*Fluencia verbal*	M = 2.80 DT = 1.48	M = 11.87 DT = 2.01	4.190	.043	.796	-28.100	118	<.001	-9.067	1.767
*Anillas*	M = 363.13 DT = 35.83	M = 147.80 DT = 23.89	19.471	<.001	.751	38.727	118	<.001	215.333	30.455

**Subtítulo:** Abreviaturas: G-DYSLEXIA = Grupo con dislexia; G-CONTROL = Grupo control; M = Media; DT = Desviación típica; F = Estadístico F (prueba de Levene/ANOVA); Sig. = Nivel de significación (*p*); η^2^ = Eta cuadrado (tamaño del efecto); t = Estadístico *t*; gl = Grados de libertad; Sig. (bilateral) = *p* de dos colas; Diferencia de medias = Diferencia entre las medias (G-DYSLEXIA − G-CONTROL); d = Tamaño del efecto de Cohen.

Los resultados muestran diferencias estadísticamente significativas entre los grupos G-DISLEXIA y G-CONTROL en varias dimensiones clave. En el perfil conductual, los niños con dislexia obtuvieron puntuaciones significativamente más altas en escalas como agresividad (M = 53.38, SD = 8.36) frente al grupo de control (M = 17.63, SD = 5.91), con un t(118) = 27.022, p < .001, un tamaño del efecto grande (d = 7.246) y una varianza explicada considerable (η^2^ = .586). De manera similar, se observaron diferencias significativas en ansiedad (t(118) = 28.417, p < .001, d = 6.990, η^2^ = .689) y depresión (t(118) = 27.134, p < .001, d = 6.964, η^2^ = .521), donde los niños con dislexia mostraron niveles elevados en comparación con sus pares sin dislexia. En cuanto a los problemas emocionales, los niños con dislexia presentaron una mayor prevalencia de depresión (M = 74.52, SD = 14.68) y ansiedad social (M = 73.63, SD = 13.48), con diferencias significativas frente al grupo de control (p < .001 para ambas), reflejando t(118) = 18.636 (d = 12.898, η^2^ = .847) y t(118) = 21.575 (d = 11.632, η^2^ = .884) respectivamente. Esto sugiere una mayor vulnerabilidad emocional en el grupo con dislexia.

Por último, en las medidas de funcionamiento ejecutivo, los niños con dislexia mostraron dificultades destacadas en la resistencia a la interferencia (M = 24.22, SD = 6.77) en comparación con el grupo de control (M = 67.62, SD = 9.66), con un t(118) = -28.494, p < .001, d = 8.342, η^2^ = .856, y en la fluidez verbal (t(118) = -28.100, p < .001, d = 1.767, η^2^ = .796). Estos resultados subrayan el impacto considerable de la dislexia no solo en las habilidades académicas, sino también en las funciones cognitivas esenciales para el aprendizaje y la adaptación social.

Por otro lado, el análisis de mediación mostró que las variables relacionadas con el diagnóstico de dislexia (problemas emocionales internalizantes, problemas de funcionamiento ejecutivo y diagnóstico de dislexia) tienen un efecto significativo sobre los problemas emocionales internalizantes [*β*=−41.41, *p*=.001], indicando una relación negativa entre el diagnóstico de dislexia y el aumento de los problemas emocionales internalizantes. Además, el funcionamiento ejecutivo también afectó significativamente los problemas emocionales internalizantes [*β*=−2.47, *p*=.01], sugiriendo que el bajo funcionamiento ejecutivo está vinculado a un incremento en los problemas emocionales internalizantes.

El funcionamiento ejecutivo, a su vez, mostró una relación significativa con el diagnóstico de dislexia. Al incluir el funcionamiento ejecutivo como variable mediadora, el efecto directo del diagnóstico de dislexia sobre los problemas emocionales internalizantes disminuyó [*β*=−4.13, *p*=.001], pero permaneció significativo, lo que indica un impacto indirecto a través del funcionamiento ejecutivo. Esto confirma que el funcionamiento ejecutivo media parcialmente la relación entre la dislexia y los síntomas internalizantes (Figura 1).

**Figura 1:** Gráfico de mediación entre dislexia, funcionamiento ejecutivo y síntomas internalizantes

Se realizó un modelo de regresión lineal múltiple para determinar los factores de dislexia que mejor predicen el riesgo de desarrollar problemas emocionales internalizantes. En este análisis, las variables independientes incluyeron el diagnóstico de dislexia y el funcionamiento ejecutivo. Los resultados obtenidos (Tabla 4) revelaron que el funcionamiento ejecutivo es uno de los predictores más fuertes para el desarrollo de problemas emocionales internalizantes.

**Tabla 4:** Resultados del modelo de regresión lineal múltiple para predecir el riesgo de problemas emocionales internalizantes

Variable	B	Error Estándar	*t*	*p*	IC 95%
Diagnóstico de dislexia	-41.41	.001	3.56	<.001	[-55.30, -30.22]
Funcionamiento ejecutivo	-2.47	.01	4.89	<.001	[-3.52, -1.42]
R^2^ ajustado	.755				

**Subtítulo:** Abreviaturas: B = coeficiente no estandarizado; Error Estándar (EE) = error típico del coeficiente; t = estadístico t; p = nivel de significación; IC 95% = intervalo de confianza al 95% para B; R^2^ ajustado = coeficiente de determinación ajustado por el número de predictores.

Los resultados muestran que tanto el diagnóstico de dislexia como el funcionamiento ejecutivo son factores significativos en la predicción del desarrollo de problemas emocionales internalizantes. El modelo indica que el 75.5% de la variabilidad en los síntomas de depresión se explica por las variables analizadas.

Del mismo modo, el análisis de mediación mostró que las variables relacionadas con el diagnóstico de dislexia (problemas de funcionamiento ejecutivo y diagnóstico de dislexia) tienen un efecto significativo sobre los problemas emocionales exteriorizados [*β*=−39.87, *p*=.001], lo que indica una relación negativa entre el diagnóstico de dislexia y el aumento de los síntomas externalizantes. Además, el funcionamiento ejecutivo también afectó significativamente los problemas emocionales exteriorizados [*β*=−3.05, *p*=.01], lo que sugiere que el bajo funcionamiento ejecutivo está vinculado a un incremento en los síntomas externalizantes.

El funcionamiento ejecutivo, a su vez, mostró una relación significativa con el diagnóstico de dislexia. Al incluir el funcionamiento ejecutivo como variable mediadora, el efecto directo del diagnóstico de dislexia sobre los problemas emocionales exteriorizados disminuyó [*β*=−5.14, *p*=.001], pero permaneció significativo, lo que indica un impacto indirecto a través del funcionamiento ejecutivo. Esto confirma que el funcionamiento ejecutivo media parcialmente la relación entre la dislexia y los problemas emocionales exteriorizados (Figura 2).

**Figura 2:** Gráfico de mediación entre dislexia, funcionamiento ejecutivo y problemas emocionales exteriorizados

Se realizó un modelo de regresión lineal múltiple para determinar los factores de dislexia que mejor predicen el riesgo de desarrollar problemas emocionales exteriorizados. En este análisis, las variables independientes incluyeron el diagnóstico de dislexia y el funcionamiento ejecutivo. Los resultados obtenidos (Tabla 5) revelaron que el funcionamiento ejecutivo es uno de los predictores más fuertes para el desarrollo de problemas emocionales exteriorizados.

**Tabla 5:** Resultados del modelo de regresión lineal múltiple para predecir el riesgo de problemas emocionales exteriorizados

Variable	B	Error Estándar	t	p	IC 95%
Diagnóstico de dislexia	-39.87	.001	3.56	<.001	[-52.80, -28.94]
Funcionamiento ejecutivo	-3.05	.01	4.89	<.001	[-4.32, -1.78]
R^2^ ajustado	.641				

**Subtítulo:** Abreviaturas: B = coeficiente no estandarizado; Error Estándar (EE) = error típico del coeficiente; t = estadístico t; p = nivel de significación; IC 95% = intervalo de confianza al 95% para B; R^2^ ajustado = coeficiente de determinación ajustado por el número de predictores.

Los resultados muestran que tanto el diagnóstico de dislexia como el funcionamiento ejecutivo son factores significativos en la predicción del desarrollo de problemas emocionales exteriorizados. El modelo indicó que el 64.1% de la variabilidad en los problemas emocionales exteriorizados se explica por las variables incluidas en el modelo.

## DISCUSIÓN

El presente estudio tuvo como objetivo explorar y comparar los perfiles conductual, emocional y de funcionamiento ejecutivo entre niños con dislexia y aquellos con un desarrollo lector normotípico. La dislexia, un trastorno específico del aprendizaje que afecta principalmente a la lectura se ha asociado tradicionalmente con dificultades académicas; sin embargo, estudios recientes han demostrado que su impacto es mucho más amplio, afectando también el bienestar emocional, el comportamiento y las funciones ejecutivas de los niños con dislexia^(44,45)^.

Uno de los hallazgos más relevantes de nuestro estudio fue la identificación de una mayor prevalencia de problemas emocionales, específicamente ansiedad y depresión, en los niños con dislexia en comparación con el resto de los participantes sin este trastorno^(46,47)^. Este resultado coincide con investigaciones previas que han mostrado que los niños con dislexia tienen un mayor riesgo de desarrollar trastornos emocionales, como la ansiedad y la depresión^(48)^, debido a las repetidas experiencias de fracaso académico y la estigmatización social^(49)^. Por ejemplo, una revisión sistemática realizada por Wilson et al.^(50)^ destacó que los niños con dislexia son significativamente más propensos a experimentar síntomas depresivos, lo que se asocia con su incapacidad para cumplir con las expectativas académicas y sociales. Esta situación puede provocar una baja autoestima y desesperanza, que refuerza los sentimientos de ansiedad y depresión. Además, estos problemas emocionales pueden llevar a una situación en la que la ansiedad y la depresión agravan aún más las dificultades de aprendizaje, creando una limitación adicional para el correcto progreso académico^(49)^. De la misma manera, es necesario considerar que la dislexia no solo afecta las habilidades académicas, sino que también tiene un impacto profundo en la salud mental de los menores^(46)^. La presión académica y la constante comparación con compañeros sin dificultades de aprendizaje pueden incrementar los niveles de estrés, afectando negativamente el bienestar emocional de estos niños^(47,48)^. Es por ello por lo que la intervención temprana y el apoyo psicológico se presentan como estrategias fundamentales para mitigar estos efectos y mejorar la calidad de vida de los niños con dislexia. Proveer un entorno de aprendizaje inclusivo y sensible a las necesidades emocionales puede reducir significativamente los niveles de ansiedad y depresión, favoreciendo un desarrollo más equilibrado en este tipo de perfiles clínicos^(50)^.

Los resultados de nuestro estudio también indicaron que los niños con dislexia muestran un mayor grado de problemas conductuales, como hiperactividad, impulsividad y conductas desafiantes, en comparación con sus pares sin dislexia. Este hallazgo es consistente con la literatura que sugiere que las dificultades de aprendizaje, como la dislexia, pueden estar asociadas con un aumento en los comportamientos disruptivos, posiblemente como una respuesta a la frustración y al estrés asociado con el bajo rendimiento académico^(51,52)^. Es importante considerar que los problemas conductuales observados en el grupo de niños con dislexia podrían ser un reflejo de su lucha interna para adaptarse a las demandas académicas que no pueden cumplir debido a sus dificultades en la lectura. Estos comportamientos pueden ser interpretados como intentos de manejar o expresar la frustración acumulada, lo que resulta en conductas desafiantes o disruptivas en el aula^(53)^. Además, la falta de comprensión por parte de los educadores y compañeros puede exacerbar estos comportamientos, creando un entorno hostil para el niño, lo que puede llevar a un aislamiento social y a un aumento de los comportamientos problemáticos^(54)^. Un aspecto interesante para considerar es que, aunque los problemas conductuales pueden ser más visibles y, por lo tanto, más fáciles de identificar, su origen subyacente en la dislexia puede ser más complejo. Las dificultades en la autorregulación emocional y en el control de los impulsos, que son características observadas en niños con dislexia, parecen estar relacionadas con déficits en las funciones ejecutivas, que dificultan la capacidad del niño para manejar sus emociones de manera adecuada^(55)^.

El funcionamiento ejecutivo es un conjunto de procesos cognitivos que incluyen la memoria de trabajo, la flexibilidad cognitiva, la planificación y la inhibición, y que son cruciales para la adaptación a las demandas académicas y sociales^(24-26)^. Nuestro estudio reveló que los niños con dislexia presentan alteraciones significativas en estas funciones en comparación con sus compañeros sin dislexia, lo que concuerda con investigaciones previas que destacan las dificultades en las funciones ejecutivas como un componente clave de la dislexia^(56,57)^. Las dificultades en las funciones ejecutivas observadas en el grupo de participantes con diagnóstico de dislexia pueden explicar en parte por qué estos niños tienen problemas para organizar y planificar sus tareas escolares, mantener la atención durante períodos prolongados y adaptarse a los cambios en las tareas^(51)^. Estas habilidades son esenciales no solo para el rendimiento académico, sino también para la vida cotidiana, ya que permiten a los individuos manejar múltiples tareas, tomar decisiones informadas y regular sus emociones^(58)^. Además, estudios neuropsicológicos han sugerido que las dificultades en las funciones ejecutivas en niños con dislexia podrían estar relacionadas con alteraciones en las redes cerebrales que soportan estas funciones^(18-22)^, especialmente en áreas como la corteza prefrontal^(59)^. Estas alteraciones podrían ser una de las razones por las cuales los niños con dislexia muestran un rendimiento inferior en tareas que requieren un control cognitivo superior, como la planificación y la inhibición^(60)^. Estos hallazgos tienen implicaciones significativas para la intervención educativa. Los programas que incluyen entrenamiento en funciones ejecutivas, como la mejora de la memoria de trabajo o la planificación, podrían ser beneficiosos para los niños con dislexia, ayudándoles a superar algunas de las barreras cognitivas que enfrentan. Además, la integración de estrategias de enseñanza que se adapten a las fortalezas y debilidades cognitivas de estos niños podría mejorar no solo su rendimiento académico, sino también su autoestima y bienestar general^(59-61)^.

Es importante señalar que muchos de los síntomas emocionales observados en los niños con dislexia pueden ser interpretados como consecuencias indirectas de sus dificultades lectoras persistentes. La frustración asociada a un desempeño académico inferior, el sentimiento de incompetencia frente a sus compañeros y la presión para alcanzar metas escolares que no están adaptadas a sus necesidades contribuyen al desarrollo de ansiedad, tristeza, desmotivación y baja autoestima. En este sentido, la dislexia no solo representa un desafío cognitivo, sino también una condición que puede desencadenar un proceso de vulnerabilidad emocional progresiva si no se identifican y abordan adecuadamente sus efectos secundarios en el entorno educativo y social.

En línea con estas observaciones, el modelo de Riesgo y Resiliencia propuesto por Hoeft et al.^(62)^ proporciona un marco conceptual valioso para comprender la complejidad de la dislexia más allá del plano académico. Este modelo plantea que, si bien las dificultades en la lectura suponen un riesgo importante para el desarrollo emocional, existen factores protectores individuales y contextuales que pueden modular sus efectos negativos. Entre estos factores se encuentran la autoestima, el apoyo familiar, las estrategias de afrontamiento y la plasticidad cerebral. Estudios de neuroimagen, como los realizados por Hoeft et al.^(62)^ han demostrado que algunos niños con dislexia logran adaptarse funcionalmente gracias a redes de compensación neuronal, lo que pone de relieve la importancia de fomentar entornos de aprendizaje positivos y resilientes. Incorporar este enfoque permite no solo entender por qué algunos niños con dislexia presentan menos sintomatología emocional que otros, sino también orientar intervenciones que fortalezcan dichas capacidades protectoras.

Por último, otro de los hallazgos de este estudio fue la identificación del funcionamiento ejecutivo como un predictor clave en el desarrollo de problemas emocionales tanto internalizantes como externalizantes en niños con dislexia. Desde una perspectiva educativa, estos hallazgos refuerzan la necesidad de diseñar programas de intervención que integren el entrenamiento en funciones ejecutivas dentro del currículo escolar. Estrategias como el uso de organizadores gráficos, rutinas visuales, técnicas de autorregulación emocional y juegos estructurados pueden facilitar la planificación, la inhibición y la flexibilidad cognitiva en los niños con dislexia. Asimismo, la implementación de adaptaciones metodológicas, como descomponer tareas complejas en pasos más manejables, ofrecer tiempos adicionales para completar las actividades o utilizar apoyos visuales, puede contribuir a un entorno de aprendizaje más accesible. Formar a los docentes en el reconocimiento de las dificultades específicas de estos niños y en el uso de herramientas pedagógicas basadas en la neuroeducación puede reducir la frustración del alumnado y fomentar su participación activa, mejorando tanto el rendimiento como el bienestar emocional en el aula. Los resultados mostraron que el diagnóstico de dislexia tiene un efecto negativo significativo sobre los problemas emocionales internalizantes. Estos resultados son consistentes con estudios previos que han demostrado que los niños con dislexia tienen un mayor riesgo de desarrollar síntomas emocionales, debido a la frustración y las dificultades persistentes en el ámbito académico^(48,63)^. Investigaciones como la de Snowling and Hulme^(64)^ han señalado que la dislexia no solo afecta las habilidades de lectura, sino que también tiene un impacto significativo en el bienestar emocional de los niños, exacerbado por las constantes experiencias de fracaso académico. El papel mediador del funcionamiento ejecutivo en esta relación es particularmente relevante. Este estudio mostró que el funcionamiento ejecutivo tiene un efecto directo sobre los problemas emocionales, tanto internalizantes como externalizantes, lo que sugiere que las deficiencias en estas funciones cognitivas agravan los síntomas emocionales en los niños con dislexia. Estos hallazgos coinciden con investigaciones previas que han mostrado que el funcionamiento ejecutivo es fundamental para la regulación emocional y el control de impulsos, lo que, a su vez, puede influir en la aparición de síntomas internalizantes, como la ansiedad y la depresión^(65)^. De hecho, Diamond^(66)^ ha argumentado que los niños con dificultades en el funcionamiento ejecutivo, como aquellos con dislexia, son más propensos a desarrollar trastornos emocionales debido a su incapacidad para gestionar el estrés académico y las demandas sociales. La reducción del efecto directo de la dislexia sobre los problemas emocionales tras incluir el funcionamiento ejecutivo como variable mediadora refuerza la importancia de este factor en la relación entre dislexia y salud mental. Este hallazgo subraya la necesidad de intervenciones que no solo se centren en las habilidades académicas de los niños con dislexia, sino también en el fortalecimiento de sus habilidades de funcionamiento ejecutivo, como una forma de mitigar el impacto emocional asociado a este trastorno^(67)^. Además, la asociación entre dislexia y problemas emocionales externalizantes, como la agresión y la conducta desafiante, ha sido ampliamente documentada en la literatura. Este estudio confirma esta relación, mostrando un efecto significativo del diagnóstico de dislexia sobre los síntomas externalizantes, lo que coincide con investigaciones que han encontrado que los niños con dificultades de aprendizaje son más propensos a presentar conductas disruptivas como una forma de gestionar su frustración y ansiedad^(68,69)^.

A pesar de los importantes hallazgos de este estudio, existen algunas limitaciones en este estudio. Una de las principales es la dependencia de autoinformes y evaluaciones realizadas por los padres, lo que podría introducir sesgos en la evaluación de los problemas emocionales y conductuales. Futuros estudios podrían beneficiarse de la inclusión de evaluaciones más objetivas, como observaciones directas en el aula o pruebas neuropsicológicas administradas por profesionales, para proporcionar una evaluación más completa y precisa. Asimismo, aunque este estudio se centró en la comparación entre niños con y sin dislexia, futuras investigaciones podrían explorar cómo factores adicionales, como el entorno familiar, la calidad del apoyo escolar y las intervenciones previas, influyen en los perfiles conductual, emocional y de funcionamiento ejecutivo de los niños con dislexia. También sería importante investigar la efectividad de diferentes programas de intervención en mejorar estos perfiles y en reducir las brechas entre los niños con y sin dislexia. Finalmente, es necesario continuar investigando las bases neurobiológicas de la dislexia y cómo estas afectan el desarrollo de funciones ejecutivas y emocionales. Comprender mejor estas relaciones podría conducir a intervenciones más personalizadas y efectivas, que no solo aborden los síntomas de la dislexia, sino que también mejoren el bienestar general y la calidad de vida de los niños con este diagnóstico.

Este estudio aporta una contribución original al evidenciar el papel mediador del funcionamiento ejecutivo en la relación entre dislexia y sintomatología emocional, lo cual ha sido escasamente explorado en la literatura en población infantil hispanohablante. Al integrar medidas conductuales, emocionales y cognitivas, este trabajo ofrece una visión comprensiva del impacto multidimensional de la dislexia. La identificación de patrones específicos entre estas variables no solo enriquece el conocimiento teórico sobre el trastorno, sino que también abre nuevas líneas de investigación e intervención para abordar de forma más holística las necesidades de los niños con dislexia en contextos escolares y clínicos.

## CONCLUSIONES

En conclusión, este estudio pone de manifiesto que los niños con dislexia presentan diferencias significativas en los perfiles emocional, conductual y de funcionamiento ejecutivo en comparación con sus pares sin dislexia. Los resultados indican que los niños con dislexia son más propensos a experimentar trastornos como la ansiedad, la depresión o problemas conductuales, como la hiperactividad y la impulsividad, lo cual se asocia con déficits en el funcionamiento ejecutivo como en la inhibición, la planificación y la memoria de trabajo. Estas dificultades no solo afectan en su rendimiento académico, sino que también tienen un impacto considerable en su adaptación social y bienestar general. Estos hallazgos subrayan la importancia de abordar la dislexia no solo desde una perspectiva académica, sino también considerando sus implicaciones emocionales y conductuales, lo que resalta la necesidad de un enfoque interdisciplinar en el estudio y comprensión de este trastorno del aprendizaje.
